# Working memory reflects vulnerability to early life adversity as a risk factor for substance use disorder in the *FKBP5* cortisol cochaperone polymorphism, rs9296158

**DOI:** 10.1371/journal.pone.0218212

**Published:** 2019-06-11

**Authors:** William R. Lovallo, Ashley Acheson, Andrew J. Cohoon, Kristen H. Sorocco, Andrea S. Vincent, Colin A. Hodgkinson, David Goldman

**Affiliations:** 1 Behavioral Sciences Laboratories, Veterans Affairs Medical Center, Oklahoma City, OK, United States of America; 2 Department of Psychiatry and Behavioral Sciences, University of Oklahoma Health Sciences Center, Oklahoma City, OK, United States of America; 3 Department of Psychiatry, University of Arkansas for Medical Sciences, Little Rock, AR, United States of America; 4 Donald W. Reynolds Department of Geriatric Medicine, University of Oklahoma Health Sciences Center, Oklahoma City, OK, United States of America; 5 Cognitive Science Research Center, University of Oklahoma, Norman, OK, United States of America; 6 Laboratory of Neurogenetics, NIH, NIAAA, Bethesda, MD, United States of America; Technion Israel Institute of Technology, ISRAEL

## Abstract

Early life adversity (ELA) negatively affects health behaviors in adulthood, but pathways from ELA exposure to behavioral outcomes are poorly understood. ELA in childhood and adolescence may translate into adult outcomes by way of modified glucocorticoid signaling. The cortisol cotransporter, FKBP5 has a G-to-A substitution (rs9296158) that hinders cortisol trafficking within target cells, and this impaired glucocorticoid signaling may shape the long-term response to ELA. We used performance on the Stroop test to assess working memory in 546 healthy young adults who had experienced 0, 1, or > 1 forms of ELA in childhood and adolescence and were genotyped for the FKBP5 rs9296158 G-to-A polymorphism. We observed a robust Gene x Environment interaction (*F* = 9.49, *p* < .0001) in which increased ELA exposure led to progressively greater Stroop interference in persons carrying AG and AA genotypes of FKBP5 with no such effect in GG carriers. Further work is needed to explore the modification of cognitive function resulting from ELA. Impairments in working memory illustrate how ELA may use glucocorticoid pathways to influence working memory with potential implications for decision-making and risky behavior including substance use disorders.

## Introduction

Exposure to early life adversity (ELA) may impair health and increase the risk for psychiatric disorders [1]. ELA exposure during childhood and adolescence affects the stress axis in early adulthood [2] and also leads to modifications of cognitive function and regulation of affect [3]. Importantly, not all persons are equally vulnerable to ELA [4, 5] suggesting that gene-by-environment (G x E) interactions may play a role in determining how ELA leads to behavioral outcomes relative to health.

With these considerations in mind, we have examined G x E relationships involving ELA exposure in the Family Health Patterns project, a broad-based study of risk factors for alcoholism incorporating psychiatric status, temperament, cognitive performance, and stress axis reactivity. We previously reported a G x E effect that demonstrated an impact of ELA on performance in the Stroop task and on heart rate reactivity to stress in healthy young adults carrying a specific polymorphism of the gene, *FKBP5*. Nevertheless, experience with G x E relationships has shown frequent failures of replication when initial reports are tested in subsequent studies [6]. Accordingly, we tested a replication sample of persons in the present study and then combined these two samples to test gene-dose effects of *FKBP5* risk alleles in relation to ELA exposure.

Cortisol is the core component of the response to stress and cortisol’s actions are targets for studying vulnerability to ELA and resulting modification of health behaviors. In addition to its role in outputs to the body, cortisol feedback to the brain acts by way of glucocorticoid receptors in the prefrontal cortex and limbic system, including hippocampus and amygdala [7–9]. Acute variations in cortisol levels therefore influence affective responses to the environment [10] and contribute to the formation of emotional memories [11]. As such, cortisol’s long-term effects depend on its ability to complex with glucocorticoid receptors for transport into the cell nucleus [12]. The glucocorticoid receptor (GR) system, including receptor availability, responsiveness, and nuclear transport, is considered a link connecting early experience to permanent modifications of approach and avoidance tendencies in adulthood, as shown in Michael Meaney’s influential studies on early experience, stress reactivity and behavioral exploration in highly nurtured or maternally deprived rats [13]. A key player in the GR pathway is the FK506 binding protein 5 (FKBP5), a molecular cochaperone essential for transporting the cortisol-GR complex into the cell nucleus where it participates in gene transcription [14, 15]. The FKBP5 protein promotes cortisol’s cellular and nuclear actions in the central nervous system [14, 16]. Although FKBP5 is necessary for cortisol transport, elevated levels may interfere with GR trafficking. In this regard, the minor, A allele of *FKBP5* (rs1360780) is more readily expressed than the major, G allele, leading to chronic elevation of FKBP5 activity that interferes with cortisol trafficking and its cellular actions in A-allele carriers [17]. FKBP5 alleles have therefore become a target for understanding individual differences in motivated behavior relative to the workings of the GR system.

The specific single nucleotide polymorphism (SNP) under study here, rs9296158, is in allelic identity and strong linkage disequilibrium with rs1360780 [17–19]. As such, rs9296158, A-allele carriers may have altered glucocorticoid regulation in the central nervous system following episodes of stress and may potentially show differential sensitivity to various forms of ELA. Several studies support this contention. For example, A-allele carriers with depressive disorders and high basal cortisol output are resistant to dexamethasone suppression [20, 21]. In relation to the current study, A-allele carriers exposed to early abusive treatment have increased symptoms of post-traumatic stress disorder [18] and proneness to suicidal behavior [19], and those exposed to emotional neglect showed elevated amygdala reactivity to emotional faces [22]. The present study explores the impact of ELA on working memory processes in carriers of the A allele of FKBP5. Working memory deficits are among a constellation of phenotypic characteristics of persons at risk for substance use disorders.

Our published finding of a G x E interaction showed that greater levels of ELA exposure led to a progressive impairment of performance on the Stroop task in persons carrying the variant, A allele of *FKBP5* [23]. Although our sample was moderately large (N = 252), as noted, previous reports of G x E effects on behaviors and health outcomes have proven difficult to replicate. Genotypes associated with behavioral outcomes are likely to exert small effects on the phenotype, and measures of the phenotype may be inexact, both of which contribute to the possibility of false positive results. Accordingly, well-powered replications of prior findings may be the most effective means of overcoming these limitations [6]. In the present paper we examined ELA effects on Stroop interference scores and stress reactivity in a replication sample of 286 young adults, and we then combined the new and older samples to examine gene-dose effects of ELA on Stroop task performance in persons carrying the AA, AG, and GG genotypes of FKBP5.

## Materials and methods

### Participants

Each subject signed an informed consent form (IRB no. 2302) approved by the Institutional Review Board of the University of Oklahoma Health Sciences Center and the VA Medical Center, Oklahoma City, Ok, USA, and was given financial compensation. Subjects were healthy young adults participating in the FHP project, a broad-based study of risk factors for alcoholism [24]. The present analysis includes 543 volunteers who were genotyped for *FKBP5* and had sufficient background data to compute ELA scores. Detailed methods have been provided elsewhere [23].

### Inclusion and exclusion criteria

Subjects were 18- to 30-year-old men and women from the local community who were in self-reported good health. Prospective volunteers were excluded if they were obese; needed prescription medications other than hormonal contraceptives; had a current medical disorder; achieved a mental age score < 22 on the Shipley Institute of Living Scale [25]; a positive urine screen for abused drugs (iCup, Instant Technologies, Norfolk, VA) or positive breath alcohol test on days of testing (AlcoMate CA2000, KHN Solutions, San Francisco). Women all had negative urine pregnancy tests at the times of testing. Smoking and smokeless tobacco use were not exclusionary. Psychiatric exclusions were: a history of alcohol or drug dependence; any substance abuse within the past 2 mo; a history of Axis I disorder, other than past depression (> 60 days prior to interview) based on the computerized diagnostic interview schedule for the Diagnostic and Statistical Manual 4th ed. [26] obtained by a computerized interview schedule (C-DIS-IV) [27].

### Assessment of early life adversity

ELA was assessed using C-DIS-IV items that covered the domains of adverse life events assessed through retrospective report by Caspi and colleagues [28, 29] as follows: Physical or Sexual Adversity (“Have you ever been mugged or threatened with a weapon or ever experienced a break-in or robbery?” “Have you ever been raped or sexually assaulted by a relative?” “Have you ever been raped or sexually assaulted by someone not related to you?”, yielding a possible 3 points) and Separation from Parents (“Before you were 15, was there a time when you did not live with your biological mother for at least 6 months?” “Before you were 15, was there a time when you did not live with your biological father for at least 6 months?”, for a possible 2 points). Each person was assigned a C-DIS-IV ELA score ranging from 0 (no events) to a maximum of 5. A subset of this study sample (N = 261) also completed the short form of the Childhood Trauma Questionnaire (CTQ-SF) [30]. The C-DIS-IV scores (0–5) were significantly correlated with CTQ-SF total scores, *r* = 0.616, *p* < .0001. Due to a limited number of C-DIS-IV reports yielding scores ≥ 3 (N = 35), the scores were recoded for further analysis to form ELA groups representing 0, 1, or > 1 events.

### Study design and procedure

Subjects passing an initial telephone contact were screened at the laboratory and then tested on 2 d for the stress protocol. Lab screening included the diagnostic interview, assessment of family history of alcoholism (FH) using family history research diagnostic criteria [24, 31], and documentation of inclusion and exclusion criteria.

#### Testing procedures

The Stroop Color-Word Test was Dodrill’s version [32] which conforms to the method used by Stroop [33]. It consists of 176 repetitions of the color words “red, orange, green, blue,” printed in a random order and in discrepant ink colors (e.g, the word “red” printed in blue ink). The subject works through the list twice, first reading the printed words and next reciting the ink colors. Time in seconds is recorded during each reading, and an interference score is calculated as the time difference between the readings, which is always greater for color recitation than for word reading. The requirement to recite the ink colors adds the additional cognitive step of suppressing the tendency to read the word itself while focusing on the color This added cognitive burden is known as the Stroop interference effect. Larger interference scores are interpreted as reflecting poorer executive functioning in working memory. Specifically, lowered response inhibition and poorer ability to direct attention to the relevant word-color attribute, and therefore higher interference scores are seen as indexing important components of working memory. Subjects also completed other tasks and self-report measures not included in this report.

Stress testing lasted 105 min, including a resting baseline (30 min) followed by simulated public speaking (30 min) and mental arithmetic (15 min) and a 30-min resting recovery. The resting control day involved sitting for 105 min over the same time of day while reading and watching nature videos. Heart rate and blood pressure were monitored every 2 min on both days using an automated monitor (Critikon, Dinamap). Stress responses were computed as the mean of the difference in respective heart rate values obtained during the stress period on the stress day relative to the corresponding values on the resting control day, as described previously [34, 35].

### Genotyping

Subjects provided a saliva sample by passive drool using an Oragene collection and preservation kit (DNA Genotek, Kanata, Ontario, Canada). DNA samples were genotyped with the Illumina OmniExpress array using standard protocols. Samples with call rates below 95% were excluded, and randomly selected samples showed an average reproducibility of 99.998%. The genotype completion rate was 0.993 (using a cutoff of 0.95 call rate).

*FKBP5* (6p21.31) is located on the short arm of chromosome 6 and spans 13 exons [36]. Several single nucleotide polymorphisms appear in close proximity, and rs1360780 has been found to be functional [37]. The OmniExpress array does not contain the rs1360780 SNP for *FKBP5*, although the linked tag SNP, rs9296158, is available and is in Hardy–Weinberg equilibrium. Prior work shows that rs9296158 is in linkage disequilibrium with rs1360780 (*r*^2^ > 0.4) (17), and so rs9296158 was used in the present analysis. We ran a quality control analysis of the first 252 samples and were able to impute 241 rs1360780 genotypes with an accuracy of 0.974 using IMPUTE2. Only three genotypes differed between rs1360780 and rs9296158; and therefore, the latter SNP was used in the present analysis because of the larger sample size.

#### Assessment of population stratification using ancestry informative marker scores (AIMS)

A panel of 2491 SNPs from the Illumina OmniExpress array was selected as AIMS based on the following criteria: (1) large differences in the reference allele frequency of pairwise SNPs from the HapMap Project between African, Chinese, and European populations; (2) mapping on different chromosomes or in different regions of the same chromosome; and (3) shared by both Illumina Human Hap550v3 and HumanOmniExpress-12v1 arrays. Individual ethnic factor scores corresponding to geographical regions: Africa, Europe, Middle East, Central Asia, Far East Asia, Oceania, and America, were estimated using STRUCTURE v2.3 software and using a known set of 1051 subjects representing 51 worldwide populations (CEPH population) as a reference (http://www.cephb.fr/en/hgdp_panel.php). The data set yielded AIMS that were predominantly of European ancestry (Table 1). The mean (SD) European AIMS of the sample was 0.83 (0.11). Seventy-nine participants had European ancestry scores of < 0.50, including 43 of African ancestry, and 9 of Native American ancestry.

**Table 1:** Demographics.

	AA/AG			GG			
**ELA**	0	1	>1	0	1	>1	
N = 543	151	75	55	150	85	27	
***Demographics***							
**Age**	23 (0.3)	23 (0.4)	24 (0.5)	24 (0.3)	24 (0.4)	24 (0.6)	
**Fem (%)**	47	61	69	52	62	67	a
**SES**	48 (1.0)	46 (1.3)	44 (1.6)	50 (1.0)	47 (1.4)	43 (2.4)	a
**Edu (yr)**	16.0 (0.2)	15.5 (0.3)	15.2 (0.2)	16.3 (0.2)	15.3 (0.3)	15.5 (0.4)	a
**AIMS (%)**	89	92	91	91	89	91	a
***Cognition***							
**MA (yr)**	18.1 (0.10)	17.6 (0.14)	17.6 (0.17)	17.9 (0.10)	18.1 (0.13)	17.4 (0.25)	
***Stress Reactivity***							
**CORT**	0.08 (.01)	0.08 (.02)	0.04 (.01)	0.11 (.04)	0.04 (.01)	0.06 (.02)	
***Substance use and BMI***							
**FH+ (%)**	18	43	69	26	45	74	
**AUDIT**	4.4 (0.3)	4.0 (0.3)	3.6 (0.4)	4.0 (0.2)	4.6 (0.4)	4.2 (0.5)	
**1**^**st**^ **Drink**	17.3 (.2)	16.8 (.4)	15.1 (.4)	16.9 (.2)	17.1 (.3)	15.4 (.7)	a
**Drugs (n)**	0.9 (.1)	1.3 (.2)	1.5 (.2)	1.1 (.1)	1.4 (.2)	1.7 (.4)	a
**Smoke (%)**	5	4	7	8	4	4	
**BMI (kg/m**^**2**^**)**	23.3 (.2)	24.0 (.3)	23.5 (.5)	23.5 (.3)	23.2 (.3)	24.1 (.7)	

**Notes.** Entries show Mean (± Std Err). F is female sex. SES is socioeconomic status based on Hollingshead’s measure of social status [38]. Edu is Education. AIMS is ancestry informative markers indicating percentage of European ancestry. MA is the Shipley Hartford Institute of Living Scale mental age score [39], estimating intellectual development in years. FH+ is the percentage of each group having a parental history of alcohol use disorders. AUDIT is the Alcohol Use Disorders Identification Test [40]. 1^st^ Drink is age at which the person reported first consuming a full drink of alcohol. Drugs is the number of recreational drugs the subject reported every trying. Smoke is the percentage of each group using cigarettes. BMI is Body Mass Index in kg/m^2^ of body surface area.

^a^ ELA groups *p*s < 0.002; Bonferroni corrected threshold for 13 tests at *p* ≤ 0.05 is 0.004

### Data analysis

In accordance with our goal of testing the reliability of our previous findings on the earlier sample of 257 persons [23], the new analysis first examined Stroop performance and heart rate reactivity scores in the new sample of 286 subjects. We then combined these samples to analyze the full sample of 543 persons. The analysis on the combined sample also allowed us to extend the earlier findings by testing gene-dose effects. Data were tested for the effects of Genotype (AA/AG, GG or AA, AG, GG), ELA (0, 1, >1), and the G x ELA interaction terms. Type III sums of squares were used to ensure independence of individual *F* ratios. In a preliminary test of the model we included FH status as a separate grouping variable and found no effects of FH or its interactions. We therefore dropped that term from subsequent tests. We also examined the impact of ancestry informative marker scores (AIMS) and sex on the G x E effects on Stroop performance by including the G x Sex, G x European AIMS, and G x African AIMS scores as covariates following the recommendation of Keller (5). Tests were considered statistically significant if *p* ≤ 0.05. Data were analyzed using SAS software, Ver. 9.2 for Windows. Copyright 2012 SAS Institute Inc. SAS and all other SAS Institute Inc. product or service names are registered trademarks of SAS Institute Inc., Cary, NC, USA. The data set analyzed in this paper is available in Open Science Framework (OSF.IO) DOI 10.17605/OSF.IO/G39QP File 2019-04-25.

## Results

Demographic and other descriptive information for AA/AG and GG allele carriers is provided in Table 1. Groups with higher levels of ELA exposure were more likely to be female and to have lower socioeconomic status scores, fewer years of education, and lower Shipley mental age scores.

### Stroop task performance

#### *FKBP5* genotype x ELA replication

**Fig 1:**
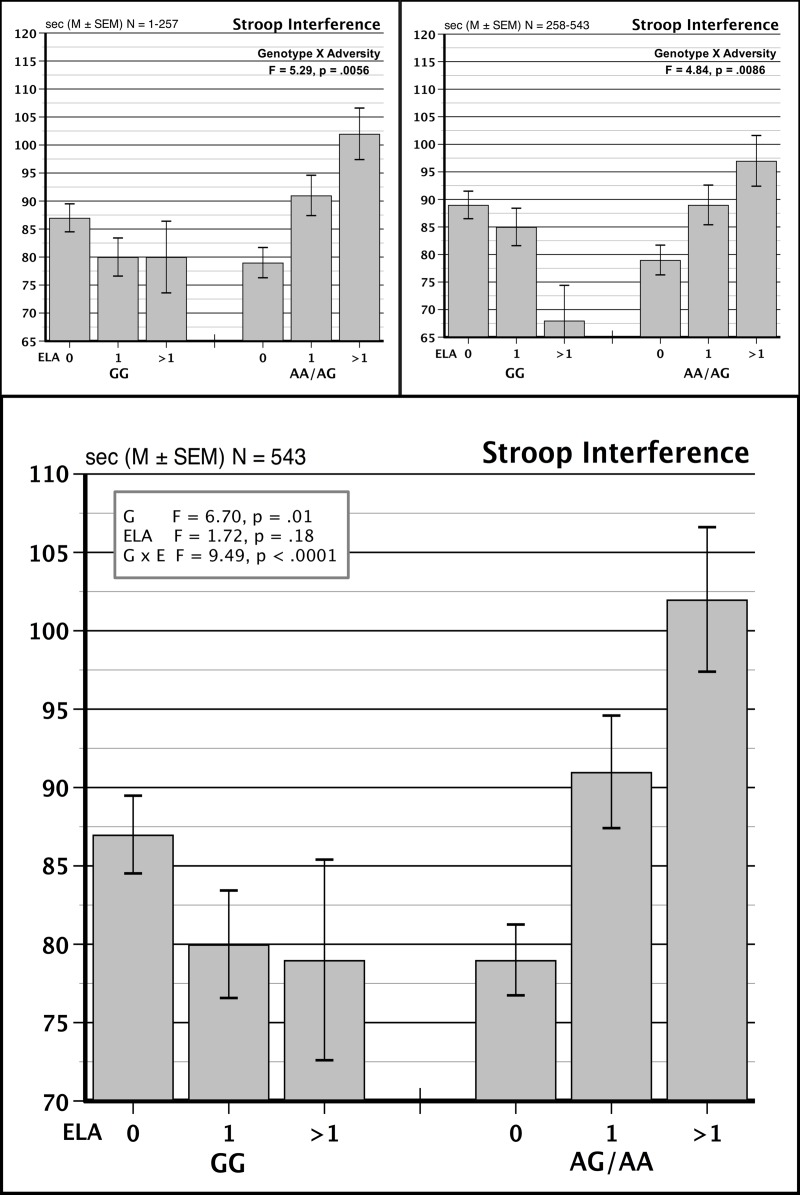
Stroop task interference scores for persons carrying GG and AG/AA genotypes of FKBP5 (rs9196158) who were exposed to 0, 1, or >1 forms of ELA during childhood and adolescence. Upper left panel shows results for the initial sample of 257 subjects previously published [23]. Upper right panel shows results for the new sample of 286 subjects. Lower panel results for the combined sample of 543 persons.

The earlier results [23] found that A-allele (AA/AG) carriers of *FKBP5* (rs9296158) exposed to 0, 1, and >1 instances of ELA had progressively greater levels of Stroop task interference, in contrast to the GG homozygotes, who showed no such effect, as seen in a significant G x E interaction, *F* = 5.29, *p* = .0056 (shown in Fig 1, upper left panel). Analysis of Stroop interference in the new subsample replicated this G x E effect, *F* = 4.84, *p* = .0086 (shown in Fig 1, upper right). The replication of our initial findings allowed us to combine the samples and to analyze of the full data set of 543 persons which also yielded a significant G x E interaction, *F* = 9.49, *p* = .0001, partial eta^2^ = .034 (Fig 1, lower panel). As an exploratory analysis, we tested the effect of ELA separately within combined AA/AG groups and for GG homozygotes and found that higher levels of ELA produced a significant increase in the magnitude of Stroop interference (*F* = 3.86, *p* = .022) while no such effect was seen within the GG homozygotes, *F* = 1.69, *p* = .186.

We tested the main G x E result in relation to potential confounders. As noted in Table 1, women reported higher levels of ELA exposure and were represented in more significant numbers than men in the 1 and > 1 ELA groups, however after the G x Sex interaction term was added to the model, the G x E interaction remained significant, *F* = 9.64, *p* < .0001. The newer subject sample contained a smaller proportion of FH+ persons than were in the earlier sample (29% vs 44%, respectively). We retested the statistical model including FH status and found no change in the reported G x E interaction, *F* = 9.32, *p* = .0001. As an added consideration, cognitive performance can be influenced by the individual’s age and years of education. Accordingly, we retested the primary model, adding Age and the Age x Genotype interaction, and found no change in the initial G x E interaction, *F* = 9.51, *p* < .0001. Similarly, the G x E effect remained after Years of Education were added to the model, *F* = 8.70, *p* = .0002. These results collectively indicate that working memory processes, measured in Stroop performance, reveal a reliable vulnerability to ELA in persons carrying the variant, one or two copies of the variant A allele of *FKBP5* (rs9296158).

#### *FKBP5* gene-dose effects

**Fig 2:**
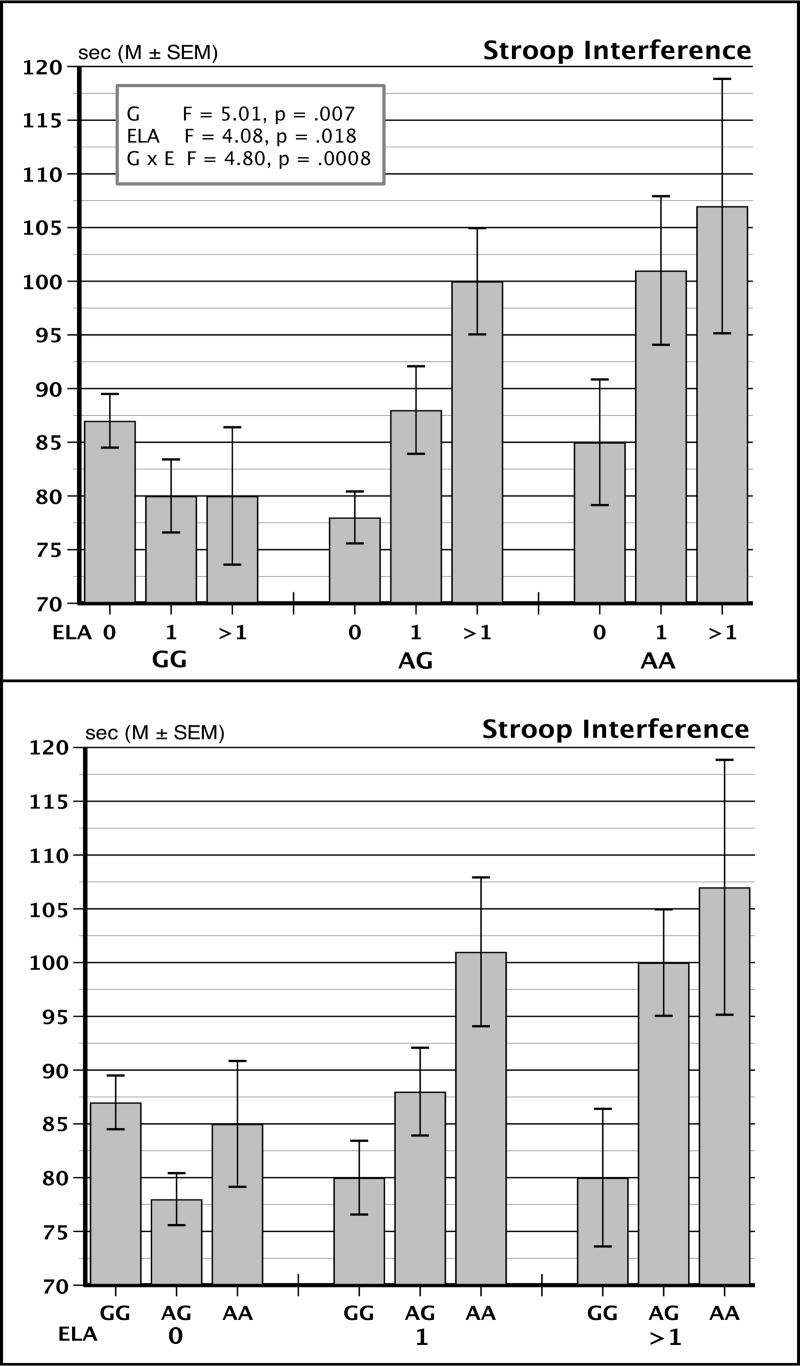
Stroop task interference scores for persons carrying GG, AG and AA genotypes of FKBP5 (rs9196158). Top panel shows results for 0, 1, or >1 ELA groups within each of the genotypes. Bottom panel shows results for the GG, AG and AA genotypes within each of the ELA groups.

The larger sample size allowed us to extend our earlier findings by examining a gene dose effect of ELA on Stroop interference scores in GG, AG, and AA allele combinations treated as separate groups. As shown in Fig 2 top panel, higher levels of ELA exposure resulted in progressively larger Stroop interference scores in both the AA- and AG-allele carriers, with no such impact in GG carriers, reflected in a significant G x ELA interaction term, *F* = 4.80, *p* = .0008. The G x E effect was not materially altered when we included the same covariates described in the previous paragraph. This interaction was accompanied by significant main effects. Stroop interference scores increased relative to the number of risk alleles, such that AA > AG > GG groups, *F* = 5.01, *p* = .007, and also increased due to higher ELA scores, *F* = 4.08, *p* = .018. For comparison purposes, the lower panel in Fig 2 rearranges the same data, illustrating that the number of A alleles has an effect on Stroop performance in the ELA = 1 and > 1 subgroups but with no effect of genotype in persons reporting 0 ELA exposure in childhood and adolescence, again illustrating the interaction of genotype and life experience.

### Heart rate reactivity to stress

HR response data for the new sample showed no effect of Genotype, *F* = 1.51, *p* = .22, ELA exposure, *F* < 1.0, or the G x E interaction, *F* < 1.0. However, given our earlier results showing an impact of ELA in A-allele carriers, we combined the two data sets to examine the full sample as shown in Table 2. The ANOVA model on the full sample also showed no significant effects of Genotype, ELA, or their G x E interaction. However, inspection of the means indicates a modestly larger effect of ELA on HR response to stress in the AA genotype group than in the other two genotype groups. We performed an exploratory *post hoc* analysis of ELA effects within each genotype and found a nonsignificant trend of ELA impact among AA carriers, *F* = 2.77, *p* = .072, and smaller differences for the AG and GG carrier groups. However, none of the AA, AG, or GG subgroups had a systematic effect of ELA exposure on HR reactivity. The effect of FKBP5 genotype and ELA on HR response to stress on the expanded sample therefore did not replicate our earlier findings [23].

**Table 2:** Heart rate reactivity.

FKBP5	ELA	Mean (SEM)	N	ELA within genotype	
**AA**				***F***	***p***
	0	12.6 (1.60)	29	2.77	0.072
	1	10.1 (1.42)	14		
	> 1	6.8 (1.54)	12		
**AG**					
	0	10.4 (0.82)	116	1.38	0.253
	1	9.0 (1.12)	61		
	> 1	7.8 (1.65)	41		
**GG**					
	0	9.3 (0.90)	146	0.01	0.995
	1	9.3 (0.95)	85		
	> 1	9.1 (1.96)	25		
**ANOVA**	***F***	***p***			
**FKBP5**	0.12	0.8860			
**ELA**	2.24	0.1074			
**FKBP x ELA**	0.63	0.6448			

## Discussion

The results replicated our earlier finding of a G x E impact of ELA exposure on executive components of working memory in carriers of the minor, A-allele, rs9296158 on *FKBP5* [23]. A-allele carriers in both the earlier and the new subsamples displayed larger Stroop interference scores than their GG-allele counterparts (Fig 1 top panels) supporting the reliability of an *FKBP5* genotype effect on components of working memory, in agreement with a study in older adults [41]. The replicated results indicate that executive function processes appear to be malleable by early experience in carriers of the *FKBP5* A allele (rs9296158).

We were also able to use the present expanded study sample to observe a gene-dose effect. ELA exerted a progressively greater impact on Stroop interference sores in relation to the presence of 0, 1, or >1 copies of the minor, A allele (Fig 2). These data also showed that *FKBP5* GG individuals were unaffected by higher levels of ELA exposure (Fig 2, lower panel) while AG and AA carriers were progressively more vulnerable to ELA. In addition, the combined sample presented here allowed us to eliminate *FKBP5* x ELA effects on heart rate responses to stress, illustrating the value of replication in candidate gene studies and the need for testing large sample sizes [6, 42].

Our data showing the impact of ELA in *FKBP5* A-allele carriers point to a vulnerability in those components of working memory tapped by the Stroop task. As noted by Smith and Jonides [43], the Stroop task is a prototypical test of working memory efficiency that addresses the person’s ability to attend to word colors and to inhibit a dominant tendency to read the words themselves. Doing so calls on sustained attention, response conflict resolution, and response selection while engaging a distal brain network involving the temporoparietal region, the anterior cingulate gyrus, and the dorsolateral prefrontal cortex [44–47]. Most pertinent to the present findings, a small study of abused adolescents, some with PTSD, showed that *FKBP5* risk-allele carriers had impaired neuronal connectivity in the brain’s temporoparietal region and in the dorsolateral prefrontal cortex [48]. These brain regions are part of a frontoparietal sustained-attention network [44] that is heavily engaged in a family of tasks including the Stroop task [47, 49]. At present, only limited attention has been paid to the effect of *FKBP5* polymorphisms on cognitive function, and to our knowledge, the FHP is the only study to examine the additional impact of ELA in this context. This paucity of such research calls for further study of specific brain mechanisms modified by ELA and their points of intersection with GR transport, targeting neuronal function in the prefrontal cortex, anterior cingulate cortex, temporoparietal region, and possibly hippocampus and amygdala. There is increasing evidence that glucocorticoid mechanisms are important in normal function of neuronal systems. We have previously reported on the acute effects of stress cortisol secretion on mental arithmetic performance, consistent with earlier findings of working memory effects in persons exhibiting natural variations in cortisol secretion [50], and we have also shown the impact of stress levels of cortisol on the startle reflex and the formation of emotional memories [11, 51]. The more limited work on *FKBP5* genotypes, is consistent with abundant evidence on widespread genomic effects of GC signaling in the central nervous system, [52] indicating a range of pathways for future study in relation to ELA.

The present study provides perspective on the actions of glucocorticoid signaling and the effects of ELA. It appears that early experience may modify adult behaviors associated with GR function both through genomic and epigenetic mechanisms. In addition to the current genotypic evidence, the influential studies of Michael Meany and colleagues in rats have called attention to epigenetic mechanisms that connect the early maternal-derived environment to later modification of GR expression and behavior, independent of the pup’s genetic background [53]. The present study suggests a genetically prepared pathway from a stressful early environment to altered cognitive function in adulthood. Others have called attention to the importance of glucocorticoid mechanisms in mediating the effects of early experience on brain function with implications for coping and emotional behavior [54].

It appears that the effect of ELA on the *FKBP5* genotype may be most apparent in behavioral dispositions and not in autonomic mechanisms. The Family Health Patterns Project has sought to test G x E models engaging phenotypic characteristics that may influence risk for alcoholism. The present results show that *FKBP5* polymorphisms can affect working memory, and working memory deficits may play a role in risky behavior associated with experimentation with alcohol and drug abuse [55]. Although our earlier Stroop task results [23] were replicated in the new subsample, we found no support for our earlier report of FKBP5 genotypes and heart rate stress reactivity. Given that this is a G x E study, the failure of replication in our HR data may reflect the small effect sizes exerted by individual genes and also the levels of systems organization through which such genes must exert their effects. It is plausible to argue that GR effects on neuronal function have a shorter pathway to the working memory phenotype than these same effects on a structure such as the heart which has multiple layers of autonomic and intrinsic regulation [56]. Small effect sizes in the phenotypic characteristics under study and the subtle effect of individual genes on behavior may account for the relatively frequent failure to replicate G x E results and the consequent need for very large sample sizes to demonstrate effects when they are present. Several others have addressed this topic with differing perspectives [4, 57–59] and Duncan and Keller provide an excellent overview [6].

There is a growing base of information on the involvement of the *FKBP5* risk allele in psychiatric morbidity. The rs9296158, A allele, in the presence of childhood abuse, has been implicated in vulnerability to PTSD, with evidence that the level of risk increases in persons exposed to several forms of early trauma [17, 60, 61]. Interactions between *FKBP5* risk alleles and ELA have also been related to a history of aggressive behavior and suicide attempts [19, 62]. However, tests of alcohol consumption have yielded contradictory results; ELA exposure and the *FKBP5* risk allele have been associated with both greater and lower consumption in adulthood [63, 64]. The present findings suggest an effect of ELA on working memory that is more severe in AA carriers than in AG and GG homozygotes. This gene dose effect is consistent with results of other studies showing cumulative effects of multiple emotional insults during childhood and adolescence. Both families of results indicate a vulnerability to the environment in A-allele carriers.

Although direct evidence is lacking, the present finding of mildly impaired executive functioning in A-allele carriers indicates a potential pathway that leads from ELA to risky decision making and impaired behavioral regulation which together may contribute to alcohol and other substance use disorders. Much evidence points to and increased risk for alcoholism in persons with impaired working memory and behavioral impulsivity [65]. Young adults with low working memory capacity are sensitive to the effects of acute alcohol administration seen in errors on a Go-NoGo test of behavioral regulation and in a wider range of tasks testing behavioral inhibition [66, 67]. Impairments of the executive function components of working memory are associated with impulsive decision making [68], and impulsive biases in decision making are prevalent in FH+ young adults and in those with antisocial temperament characteristics [69]. FH+ persons display unusual patterns of activity of the prefrontal cortex and in temporoparietal regions during working memory tasks including the Stroop task [47, 70]. Executive function impairments are associated with poor delay of gratification as seen in rapid discounting of delayed rewards [71], an effect that occurs in persons with a family history of alcoholism [72], in recreational drug users [73], and persons exposed to ELA [74]. Impaired working memory, rapid delay discounting, and impulsive decision making are worsened by exposure to ELA and are seen as behavioral phenotypes that contribute to risk for addictions [24, 75, 76]. These clinical findings point to a need for further study of intermediate phenotypes representing G x E interactions, including endocrine, neurochemical, and brain structural characteristics of *FKBP5* A-allele carriers.

In considering the impact of ELA, alone and in relation to *FKBP5* genotype, we note that the levels of ELA reported by our volunteers are nontraumatizing, a function of the FHP selection criteria which sought to test only psychiatrically healthy persons and those who were unaffected by significant use of alcohol or drug exposure. Accordingly, the sample was restricted to a nonabusing range of alcohol intake and recreational drug use and limited to persons without significant psychiatric morbidity. Therefore, the results indicate that nontraumatizing ELA experienced by otherwise healthy carriers of the *FKBP5* A allele (rs9296158) may nonetheless have a measurable decline in executive function processes. The conditions specified for study inclusion emphasized risk factors for alcoholism in the absence of secondary consequences of alcohol exposure. This also suggests that the study of ELA and *FKBP5* polymorphisms should be extended to groups with a greater range of alcohol and drug intake histories and more severe levels of ELA. Finally, the test of cognition used here is one of many that are available. The replication of our initial findings also suggests that cognitive processes may merit use of a wider range of working memory tasks engaging other components of executive function.

## Conclusions

Persons carrying one or two copies of the variant, A allele of the molecular cochaperone protein, FKBP5 appear to be vulnerable to impact of ELA on executive functions, while their GG-allele counterparts appear to be unaffected. Impaired cellular trafficking of cortisol in A-allele carriers may illustrate a point of systems response to ELA that exerts a deleterious effect in adulthood. This G x E effect appeared here as a modest impairment of working memory performance, thereby implicating glucocorticoid actions in working memory systems, including the prefrontal cortex and the temporoparietal region, as targets for study in A-allele carriers exposed to ELA during childhood and adolescence. Further work is needed to understand the molecular and cellular points of intersection connecting ELA with glucocorticoid mechanisms that affect the central nervous system.
